# Transcriptomic response and immunological responses to chimpanzee adenovirus- and MVA viral-vectored vaccines for RSV in healthy adults

**DOI:** 10.1093/cei/uxad003

**Published:** 2023-01-09

**Authors:** C Green, J McGinley, C Sande, S Capone, S Makvandi-Nejad, A Vitelli, L Silva-Reyes, S Bibi, C Otasowie, D Sheerin, A Thompson, C Dold, P Klenerman, E Barnes, L Dorrell, C Rollier, A Pollard, D O’Connor

**Affiliations:** Oxford Vaccine Group, Department of Paediatrics and the NIHR Oxford Biomedical Research Centre, University of Oxford, Oxford, UK; Institute of Microbiology & Infection, University of Birmingham, Birmingham, UK; Oxford Vaccine Group, Department of Paediatrics and the NIHR Oxford Biomedical Research Centre, University of Oxford, Oxford, UK; Oxford Vaccine Group, Department of Paediatrics and the NIHR Oxford Biomedical Research Centre, University of Oxford, Oxford, UK; Experimental Vaccinology Department, ReiThera Srl, Roma, Italy; Nuffield Department of Medicine, University of Oxford NDM Research Building, Oxford, UK; Experimental Vaccinology Department, ReiThera Srl, Roma, Italy; Oxford Vaccine Group, Department of Paediatrics and the NIHR Oxford Biomedical Research Centre, University of Oxford, Oxford, UK; Oxford Vaccine Group, Department of Paediatrics and the NIHR Oxford Biomedical Research Centre, University of Oxford, Oxford, UK; Oxford Vaccine Group, Department of Paediatrics and the NIHR Oxford Biomedical Research Centre, University of Oxford, Oxford, UK; Oxford Vaccine Group, Department of Paediatrics and the NIHR Oxford Biomedical Research Centre, University of Oxford, Oxford, UK; Oxford Vaccine Group, Department of Paediatrics and the NIHR Oxford Biomedical Research Centre, University of Oxford, Oxford, UK; Oxford Vaccine Group, Department of Paediatrics and the NIHR Oxford Biomedical Research Centre, University of Oxford, Oxford, UK; Experimental Medicine Division, Nuffield Department of Medicine, University of Oxford, Oxford, UK; Experimental Medicine Division, Nuffield Department of Medicine, University of Oxford, Oxford, UK; Nuffield Department of Medicine, University of Oxford NDM Research Building, Oxford, UK; Oxford Vaccine Group, Department of Paediatrics and the NIHR Oxford Biomedical Research Centre, University of Oxford, Oxford, UK; Oxford Vaccine Group, Department of Paediatrics and the NIHR Oxford Biomedical Research Centre, University of Oxford, Oxford, UK; Oxford Vaccine Group, Department of Paediatrics and the NIHR Oxford Biomedical Research Centre, University of Oxford, Oxford, UK

**Keywords:** RSV, vaccine, transcriptomics, human trials, antibodies, viral vectors

## Abstract

Cohorts of healthy younger adults (18–50yrs) and healthy older adults (60–75yrs) were immunized intramuscularly or intranasally with an adenovirus-vectored RSV vaccine (PanAd3-RSV) as a prime dose and boosted with PanAd3-RSV or a poxvirus-vectored vaccine (MVA-RSV) encoding the same insert. Whole blood gene expression was measured at baseline, 3- and 7-days post vaccination. Intramuscular prime vaccination with PanAd3-RSV induced differential expression of 643 genes (DEGs, FDR < 0.05). Intranasal prime vaccination with PanAd3-RSV did not induce any differentially expressed genes (DEGs) in blood samples at 3 days post vaccination. Intranasally primed participants showed greater numbers of DEGS on boosting than intramuscularly primed participants. The most highly enriched biological processes related to DEGs after both prime and boost vaccination were type-1 interferon related pathways, lymphocytic and humoral immune responses.

## Teaser

PanAd3 vaccination of healthy adults induces type-1 interferon and humoral immune responses.

## Graphical Abstract

**Graphical Abstract:**
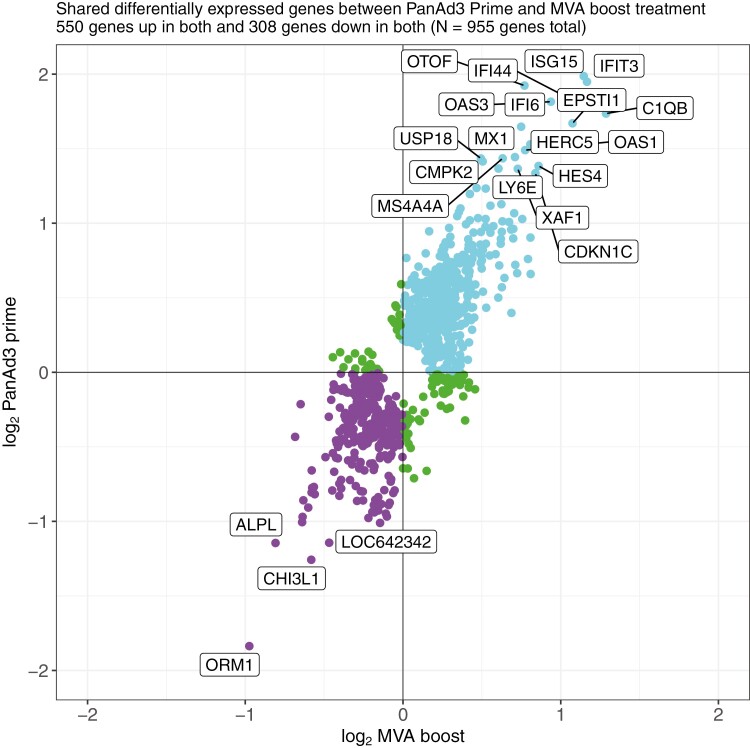

## Introduction

Viral vectors that deliver gene products from unrelated organisms are one of the leading platforms for the development of novel vaccines [1] and are capable of stimulating both antigen-specific antibody and cellular immune responses [2]. This provides the potential for control of vaccine-preventable diseases of global importance and outbreak pathogens that threaten health security. Significant progress has been made in development and testing of viral vectored vaccines for diseases such as malaria, tuberculosis, HIV, influenza, rabies, hepatitis C, and group B Meningococcus [3–10], and the flexibility of their design means that this approach can be used to rapidly develop new vaccines to control mycobacterial and viral epidemics, as recently demonstrated for Ebola in West Africa [11–13], and now in response to the COVID-19 pandemic [2, 14]. Several viral vectors have been tested in humans, including Rhabdovirus (vesicular stomatitis virus, modified vaccinia virus Ankara), alphavirus, human adenovirus (Ad5), and chimpanzee adenovirus (ChAd3, ChAd63, ChAdOx1, ChAd155, PanAd3, ad6, ad25, ad35, GAd20, and GrAd32), and these biological platforms have been shown to have good safety profiles in trials thus far and are immunogenic [2]. Efficacy of these platforms has been well characterized in recent years through the success of INN- Ad26.COV2-S and Vaxzevria vaccines in recent years. Understanding the underlying biological pathways that lead to the generation of desirable immunity could profoundly inform the design of optimal vectors, inserts, or the development of entirely novel technologies [15], particularly in light of the ongoing COVID-19 pandemic, where at the time of writing 13 of the 44 candidate vaccines currently in trials use viral vectors of some sort. Interrogation of early pathways induced by vaccination through analysis of gene expression, and linking early response patterns to immunological endpoints has led to the successful identification of key determinants of vaccine immunogenicity for several viral and subunit vaccines [16].

Analysis is reported here of the early host transcriptomic responses to two novel respiratory syncytial virus (RSV) viral-vectored vaccines in humans (PanAd3-RSV and MVA-RSV) encoding the same three antigens (F0ΔTM, N, and M2-1 proteins). In this study, participants were given PanAd3-RSV either by intramuscular or intra-nasal prime followed by homologous or heterologous boost, allowing insight into the host response to the same vaccine given by a different route, and to the same antigen delivered by a different viral vector. The aims of this study were to identify a transcriptomic signature in response to vaccination, and demonstrate which elements of this signature are linked to generation of desirable neutralizing antibody responses. The safety and immunogenicity of this vaccine have been previously assessed [17], the present study seeks to characterise the transcriptomic response to the vaccine and relate it to the results of the previous study.

## Materials and methods

### Volunteers/clinical trial

The RSV001 clinical trial was an open-label, dose-escalation, phase one evaluation of safety and immunogenicity of four combinations of prime/boost PanAd3-RSV and MVA-RSV vaccine, using combinations of vaccine in 42 healthy adults aged 18–50 years, with the primary findings from the study previously published [17]. Volunteers were randomized into four groups and were primed with either intramuscular (IM) or intra-nasal (IN) PanAd3-RSV and 4- or 8-weeks later boosted with either IM PanAd3-RSV or IM MVA-RSV (Table 1). A further group of 18 older adults aged 65–75 years was given similar combinations of prime and boost vaccines, with an additional naïve group of 6 participants [18]. A full description of all treatments given to each group has been described in a previous publication [17].

**Table 1.** Overview of study design and demographics

Group	Age group	Number enrolled	Median age	Number male	Prime vaccination	Boost vaccination (week 4)	Boost vaccination (week 8)
1	YA	11	24 (19–42)	5 (45%)	IM PanAd3-RSV	NA	IM MVA-RSV
2	YA	10	27.5 (22–48)	5 (50%)	IM PanAd3-RSV	IM PanAd3-RSV	NA
3	YA	10	28 (19–41)	6 (60%)	IN PanAd3-RSV	NA	IM MVA-RSV
4	YA	11	27 (19–48)	7 (64%)	IN PanAd3-RSV	NA	IM PanAd3-RSV
5	OA	6	68 (64–74)	3 (50%)	IM PanAd3-RSV	IM PanAd3-RSV	NA
6	OA	6	71 (65–72)	3 (50%)	IN PanAd3-RSV	NA	IM MVA-RSV
7	OA	6	67.5 (65–72)	4 (68%)	IM PanAd3-RSV	NA	IM MVA-RSV

Volunteers were healthy adults aged 18–50 years (YA) or 65–75 years (OA) who gave written informed consent to enter the trial, which included the analysis of host genetic factors. The eligibility criteria, screening, and selection of volunteers is described in detail in published material [18]. Note the terms ‘prime’ and boost’ used by convention from studies in antigen-naïve populations, but for RSV all volunteers have natural immunity from previous exposure and the terms are used in relation to the first and second dose of vaccine.

### Gene expression analyses

Gene expression was determined using Illumina Human HT-12 v4 bead chips (Illumina Inc). Samples were randomised across beadchips to minimize clustering of volunteers or time-points after vaccination by batch effect. The fluorescent intensity values were scanned with an Illumina iScan System by the Wellcome Centre for Human Genetics core facility (Oxford, UK), and raw data were extracted and formatted using GenomeStudio software.

### Identification of differentially expressed probes after vaccination

Linear regression with an empirical Bayes model was applied to the expression value of each probe after prime and boost with adjustment for volunteer age, sex, vaccine dose, day of sample collection and sample scan date using the ‘*lmfit’* and ‘eBayes’ functions of the limma package [19]. The duplicate correlation option was applied to account for the paired sampling and arrays were weighted by quality score [20, 21]. A log2 fold change of 0.32 (absolute fold change ≥1.25) from pre-vaccination baseline combined with a 5% false discovery rate (FDR) adjusted *P*-value of <0.05 were used as criteria for the identification of differentially expressed probes.

### Gene enrichment analysis

Ranked gene set enrichment analysis (GSEA) using the GSEA algorithm developed by the broad institute [22] was run on all 25 754 detected probes on the array chip for 3 and 7 days post intramuscular prime vaccination and MVA boost vaccination in all participants using the c7 database for immunological terms, and the c5 databases for biological processes (BP) and molecular function (MF).

## Results

In this study, whole blood samples were analysed from participants before, 3 days (early) and 7 days (late) after vaccination with IM or IN PanAd-RSV and following heterologous or homologous IM boost.

### Blood transcriptome perturbed by intramuscular prime vaccination but not by intranasal prime vaccination

**Figure 1.**
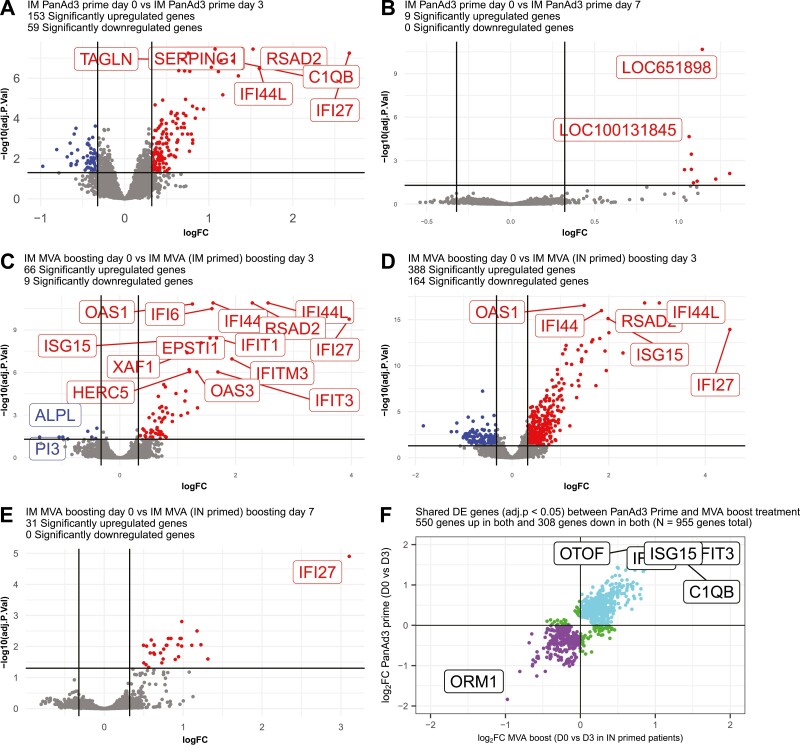
Volcano plot illustrations of early transcriptional responses to RSV vaccination. **A**: Transcriptional response of *N* = 21 volunteers who received PanAd3-RSV prime vaccination by IM injection measured 3-days after vaccination. B: Transcriptional response of *N* = 21 volunteers who received PanAd3-RSV prime vaccination by IM injection measured 7 days after vaccination. **C**: Transcriptional response of *N* = 11 volunteers who received MVA-RSV boost vaccination by IM injection after being primed with IM PanAd3-RSV measured 3 days after boost vaccination. **D**: Transcriptional response of *N* = 10 volunteers who received MVA-RSV boost vaccination by IM injection after IN PanAd3 prime vaccination measured 3-days after boost vaccination. **E**: Transcriptional response *N* = 10 volunteers who received MVA-RSV boost vaccination by IM injection after IN priming with PanAd3-RSV measured 7 days after boost vaccination. Red dots denote significantly differentially expressed probes (FDR < 0.05, log2 fold change > 0.26), blue dots significantly differentially expressed probes with fold change < 1.2, green dots indicate genes with a log2 fold change > 0.26 but which are not significantly differentially expressed. Horizontal dotted lines represent FDR cutoffs, vertical dotted lines represent log2 fold change cutoffs. (**F**) Agreement plot of differentially expressed probes 3 days after IM PanAd3-RSV prime or IM MVA-RSV boost vaccination (IN primed volunteers). Plot of the log2 adjusted fold changes of all shared differentially expressed genes 3 days after intramuscular priming with PanAd3-RSV and intramuscular boosting with MVA-RSV in participants primed intramuscularly. Points coloured in purple represent genes that are downregulated post both vaccinations. Blue points are genes that are upregulated after both vaccinations. No differentially expressed genes were downregulated in response to one vaccination and upregulated in response to another.

Compared with baseline, a total of 212 genes (Fig. 1A) were differentially expressed between baseline and 3 days following IM PanAd3-RSV prime (groups one and two combined, due to identical prime regimens). One hundred and fifty three genes were up-regulated and 59 genes were down-regulated at this time-point. No genes were found to be differentially expressed at 3 days post intranasal prime with PanAd3 RSV (groups three and four combined, FDR < 0.05).

### Blood transcriptome profile associated with plasma cells seen 7 days post intramuscular prime vaccination

Compared with baseline, a total of 9 genes were found to be differentially expressed between baseline and 7 days following IM PanAd3-RSV prime (study groups one and two combined due to same prime vaccination, Fig 1). None of these genes were down-regulated and 9 were up-regulated (Fig 1B). No genes were found to be differentially expressed 7 days following IN PanAd3-RSV prime vaccination (groups three and four combined).

**Figure 2.**
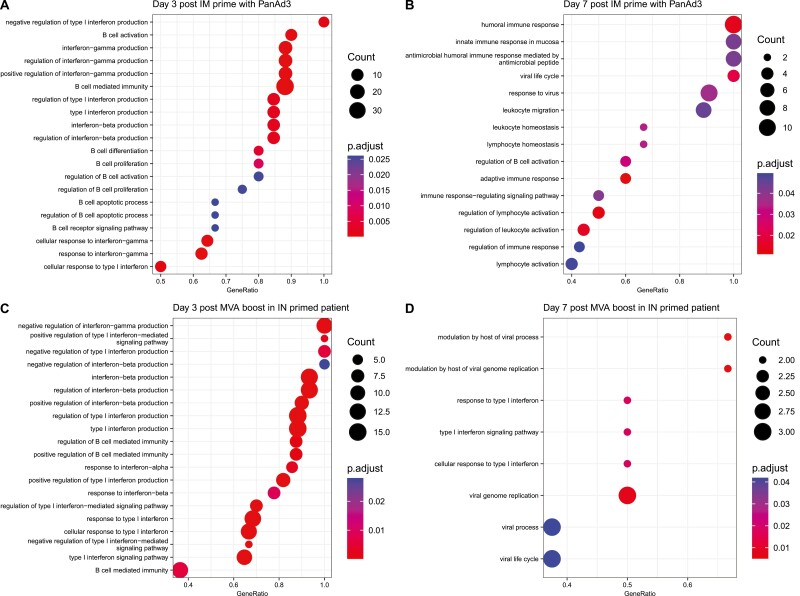
Gene set enrichment analysis of biological processes of the response to prime vaccination three and seven days after vaccination. Gene enrichment analysis for terms relating to biological processes was performed on the ranked list of all probes on the array at 3 (**A**) and 7 days (**B**) post prime vaccination and 3 (**C**) and 7 days (**D**) post boost vaccination by MVA-RSV in participants primed intranasally. Most significantly enriched terms are ranked by enrichment score. Dots are coloured based on the FDR of the associated term. Size of dots is based on the number of genes associated with that term. FDR <0.05 was used. Early transcriptional changes correlate with vaccine responses in younger adults

In order to further interpret the perturbation of the whole blood transcriptome following prime and boost vaccination, a gene set enrichment analysis was performed on all genes on the array ranked by their t-statistic using databases for gene ontology (GO) biological process terms (Fig 2A–D), GO molecular function terms (Supplementary Fig. S2A–D), and immunological terms (Supplementary Fig. S3A-D). Terms that were significantly enriched (FDR < 0.05) were represented and ranked according to the enrichment score of the term. The most enriched gene sets 3 days post prime vaccination (Fig. 2A) were type I interferon response genes, with additional general anti-viral responses, alongside B cell responses. The most enriched terms at 3 days post boost vaccination (Fig. 2C) were similar, with many terms representing type I interferon responses, B cell responses, and general anti-viral responses. Gene set enrichment analyses were also performed on the ranked lists of genes at later time points post vaccination. B-cell and plasma cell-related gene expression were seen at these time points using the immunological database (Supplementary Fig. S3B, D).

### IM MVA-RSV boost induces early transcriptional responses similar to IM PanAd-RSV prime in intranasally primed participants

A total of 75 genes (Fig. 1C) were differentially expressed 3 days after boost vaccination with MVA-RSV in participants primed intramuscularly with PanAd3-RSV. Sixty-six of these were up-regulated and 9 were down-regulated. A total of 552 genes (Fig. 1D) were differentially expressed 3 days after boost vaccination with MVA-RSV in participants primed intranasally with PanAd3-RSV. Three hundred and eighty-eight of these were up-regulated and 164 were down-regulated. Overall a total of 955 genes were commonly differentially expressed following IM PanAd3-RSV prime and IM MVA-RSV boost 3 days post vaccination in intranasally primed participants (Fig. 1F). All genes that are differentially expressed in both vaccinations are regulated in the same direction (Fig. 1F).

Thirty-one genes were found to be differentially expressed 7 days post boost vaccination in patients primed with IM-PanAd3-RSV and boosted with IM-MVA-RSV (Fig. 1E), all of which were up-regulated. Only two genes were differentially expressed 3 days post vaccination in participants both primed and boosted with IM-PanAd3 RSV. In patients primed with IN-PanAd3-RSV and boosted with IM-PanAd3-RSV only one gene was found to be differentially expressed 3 days post boost vaccination.

Changes in gene expression at 3 days post IM prime vaccination and 3 days post boost vaccination (for both priming routes) were found to have statistical correlations with the change in key measures of RSV immunity in the blood of vaccine recipients (*p* < 0.05), in particular the expansion of F-specific IFN-γ T-cells (14 days after prime, 7 days after boost), the appearance of F-specific IgG and IgA antibody secreting cells 7 days after vaccination, and with changes in vector-specific and total-RSV neutralizing antibody titres 28 days after vaccination (Table 2). These neutralizing antibody data have been previously published [18]. Forty-three genes differentially expressed 3 days post IM prime correlated (either positively or negatively) with the fold changes in neutralizing antibody 28 days post prime vaccination (Table 2), 15 genes with the fold change expansion of F-specific IFNγ producing T cells 14 days post prime vaccination (Table 2), four genes correlated with PanAd3 (vector)-specific serum neutralizing antibody titres 28 days post vaccination (Table 2), and 23 and 20 genes correlated with the number of antibody secreting cells producing IgG and IgA in response to RSV F protein 7 days post vaccination, respectively (Table 2). Two hundred fifty-five were also shown to have correlations with immunological outcome measures seven days post vaccination, despite the lack of differential expression at this time point (Table 2).

**Table 2.** Differentially expressed probes 3 and 7 days after IM PanAd3-RSV prime vaccination with significant statistical correlation to clinical/immunological responses measured at later time points

Vaccination	Number of genes correlated with PRNT 28 days post vaccination	Number of genes correlated with IFNγ 14 days post vaccination	Number of genes correlated with VnAb 7 days post vaccination	Number of genes correlated with ASC-G 7 days post vaccination	Number of genes correlated with ASC-A 7 days post vaccination
3 Days post PanAd3 priming	43	15	4	23	20
3 days post MVA boost (IM PanAd3 primed)	6	0	NA	0	0
3 days post MVA boost (IN PanAd3 primed)	32	20	NA	9	6
7 Days post PanAd3 prime	42	11	85	73	44
7 days post MVA boost (IM PanAd3 primed)	15	57	18	94	23
7 days post MVA boost (IN PanAd3 primed)	12	53	51	19	31

Data from volunteers in study groups one, two, three, and four combined (*n* = 42). The significant correlations (Pearsons *P*-value 0.05) between changes in gene expression from the 634 of differentially expressed probes 3 days after IM PanAd3-RSV prime and the 782 and 94 probes, respectively differentially expressed after boost vaccination with MVA-RSV after either intramuscular or intranasal priming and log_2_-transformed fold-change in serum anti-PanAd3 vector neutralising antibody 4-weeks after vaccination (VnAb), log_2_-transformed fold-change in serum RSV-neutralising antibody titre 28 days after vaccination (PRNT), log_2_-transformed anti-F IgG and IgA antibody secreting cell count 7 days after vaccination (ASC-G, ASC-A), and the log_2_-transformed fold-change in IFNγ count 14 days after prime.

Participants who had been primed intramuscularly with PanAd3 and then boosted with MVA elicited similar responses, with 33 genes differentially expressed 3 days post boost that correlated with neutralising antibody 28 days post boost vaccination(Table 2), 20 genes were correlated with the magnitude of F-specific IFNγ producing T-cells 7 days post boost (Table 2), and nine and six genes correlated with the number of antibody-secreting cells producing IgG and IgA at seven days post vaccination in response to RSV F protein, respectively (Table 2). Boosting with IM PanAd3 after intranasal vaccination with IN PanAd3 RSV induced no significant differential expression (not shown).

### Early gene regulation replicated in older adults but did not correlate with vaccine responses

**Figure 3.**
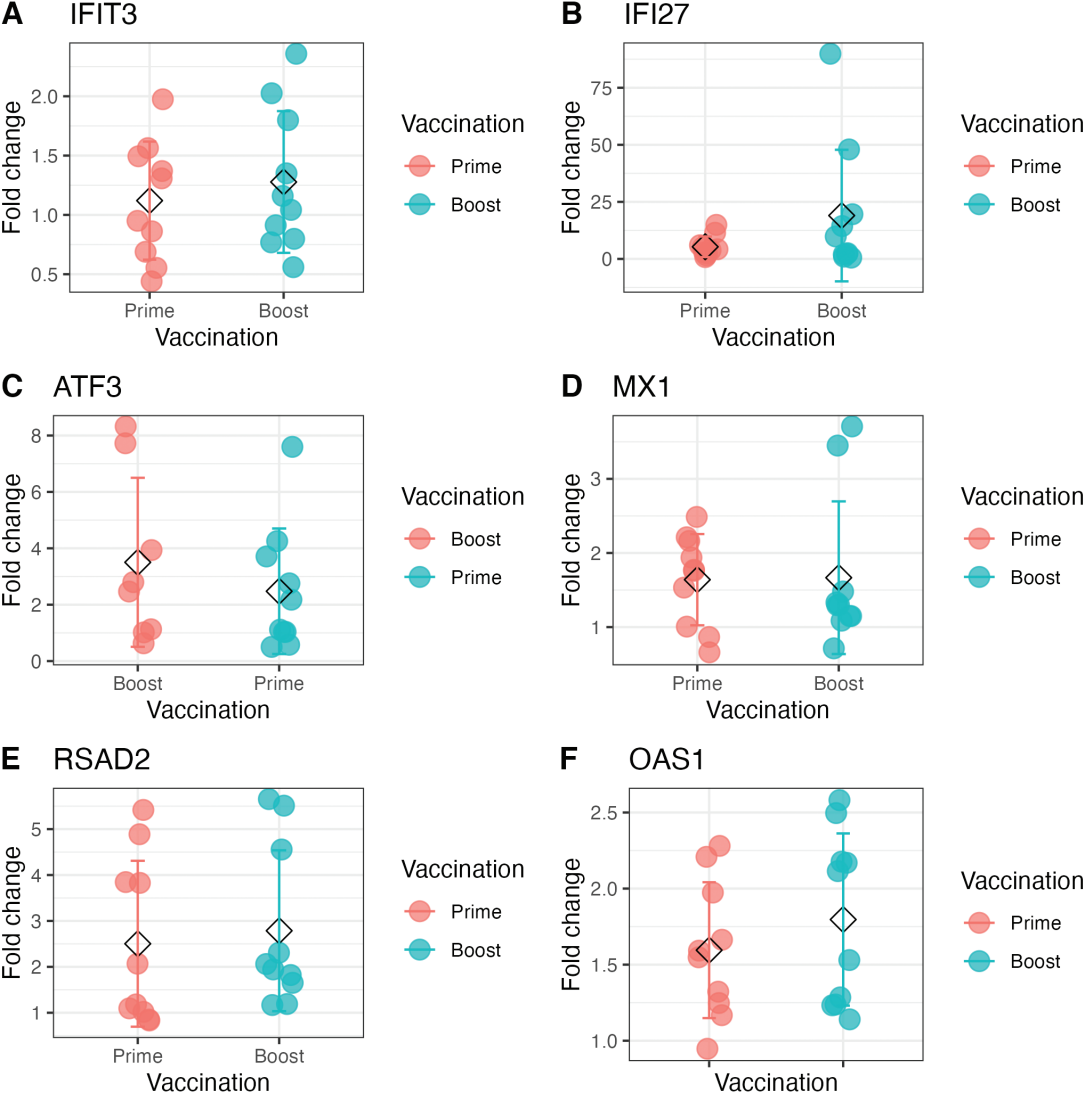
(**A**–**F**) IFI27, MX1, OAS1, and RSAD2 are significantly differentially expressed in older adult grouping after prime vaccination with PanAd Titres of genes determined to be relevant to the generation of immunological outcomes such as neutralising antibody response were measured in older adults by qPCR. Results are presented as ddCT between day of vaccination and 28 days post vaccination. Asterisk indicates differential expression of that gene compared with baseline levels (*P* < 0.05), genes after prime vaccination are coloured in red while genes after boost are coloured in blue.

**Figure 4:**
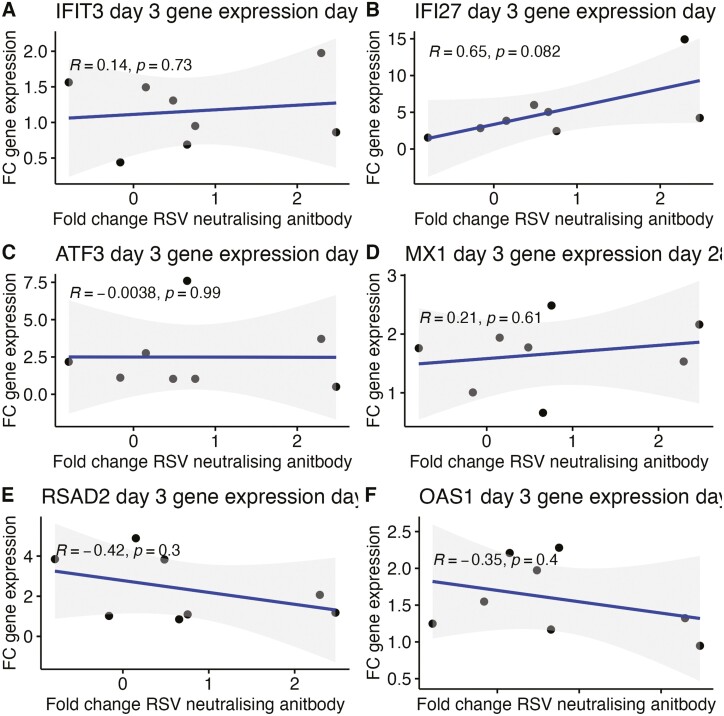
(**A**–**F**) Correlation between gene expression and neutralising antibody in older adults Pearson correlation between changes in gene expression 3 days post prime vaccination in older adults as measured by rt-qPCR and log 2-fold change in RSV neutralizing antibody measured at 28 days post vaccination. Grey area indicates confidence interval for the data. Line represents degree of correlation between fold change in gene expression and fold change in neutralizing antibody.

A further cohort of older adults was vaccinated with PanAd3 RSV and MVA RSV (Table 1). Gene expression analysis with RT-qPCR was performed in this group to analyse the expression of genes found to be of interest based on their relationship with immunological outcomes in younger adults. Six genes were selected for differential expression analysis in older adults. These genes were chosen as they had the most significant correlations with neutralizing antibody responses in older adults from transcriptomic data. Of the genes selected for differential expression analysis by RT-qPCR, *IFI27*, *MX1*, *OAS1*, and *RSAD2* were found to be differentially expressed, while *IFIT3* and *ATF3* were not found to be expressed in older adults (fig. 3A–F). After boost vaccination with MVA-RSV, *ATF3*, *IFI27*, *MX1*, *OAS1*, and *RSAD2* were found to be differentially expressed, while *IFIT3* was not found to be significantly differentially expressed, but none of these genes correlated with changes in neutralising antibody in older adults (Fig. 4A–F). Gene expression analysis by RT-qPCR was not possible in younger adult samples as these had been fully depleted before commencement of RT-qPCR experiments.

## Discussion

In this whole-blood gene expression analysis, we found a rich repertoire of 634 differentially expressed genes 3 days after intramuscular prime vaccination with PanAd3-RSV, and 94 differentially expressed genes 3 days after subsequent boost vaccination with MVA-RSV. Intranasal prime vaccination with PanAd3-RSV did not induce differential gene expression in blood, but did induce differential expression after boosting. Seven hundred and eighty-two genes differentially expressed genes were detected 3 days after MVA-RSV boost, suggesting that either there was an effect from the intra-nasal route of priming that could not be appreciated in blood or MVA boosting results in greater gene expression where participants have not been intramuscularly primed. In younger adults, the magnitude of expression of several genes correlated with the immunological responses to vaccination measured at later time points, suggesting some linkage between expression of these genes and the development of neutralizing antibody responses.

One notable gene was *IFI27*, which had the greatest fold-changes in induction after IM PanAd3-RSV and IM MVA-RSV 3 days post vaccination. *IFI27* expression is consistently observed in RSV infection with a high degree of consistency between RSV-specific signatures derived from PBMCs, whole blood, and airway epithelial cells [23]. *IFI27*, together with other genes that were differentially expressed, such as *IFI44*, *OAS3*, *EPSTII*, and *IFI44L*, are generally significantly up-regulated in whole blood from infants admitted to hospital with severe RSV-bronchiolitis [24]. These data indicate that several genes are differentially expressed following vaccination and are representative of the host response to adenoviral vector vaccines, and are likely to have had biologically relevant roles in the host response to these viral vectors and/or the RSV gene inserts in the vector. The fact that strong and consistent expression of interferon markers is seen after vaccination is a promising indicator of efficacy, as these responses have been linked to longer and more effective protection in viral vectored vaccination previously, and serve as a promising indicator of immunogenicity.

The PanAd3 vector applied by intra-nasal spray (used as an influenza vaccine in mice and RSV vaccine in rodents and new-born calves) induced immune responses in the lungs and spleen without any notable changes in immunity measured in blood that were protective to experimental challenges [25–27]. Notably, the live-attenuated IN flu vaccine (LAIV, FluMist) protects from infection despite a relative paucity of responses measurable in blood [28, 29]. RSV-specific immune responses to IN PanAd3-RSV prime in humans were equally absent or minimal in blood, but these responses were inducible with the first dose of IM vaccine at boost (IM PanAd3-RSV or IM MVA-RSV). Of particular interest was the absence of expected transcriptional responses to the boosting with IM PanAd3-RSV (after IN prime) when compared with the greater number of differentially expressed probes induced following intramuscular boosting after intranasal priming. These observations, combined with the gene set enrichment analysis, indicated a subtle response to mucosal immunisation with potential to alter the blood transcriptomic response to an IM booster vaccine. Similar to what was observed here, the injectable inactivated influenza vaccine (TIV) induced the expression of interferon genes shortly after vaccination. In contrast, intra-nasal live-attenuated influenza vaccine (LAIV) induced interferon gene expression 7 days after vaccination and only in children less than 5-years old [30].

While our analyses showed a modest number of differentially expressed genes seven days post prime vaccination, with PanAd3-RSV and boost vaccination with MVA-RSV, ranked gene set enrichment analysis (ranked using *t*-statistics for differential gene expression) of the entire probeset showed enrichment for a number of terms related to the development of memory CD4+ and CD8+ T-cell responses and humoral immunity at these time points. The development of these memory and humoral immune responses seven days post vaccination with a viral vector vaccine has been previously observed, and is consistent with what is accepted and desirable for these vaccinations [31]. Several genes correlated with clinical and antigen-specific immune responses, giving an indication of potential pathways that lead towards safe and durable protective immunity, however these genes may not necessarily reflect the process by which these immune responses are generated in at risk groups such as older adults. It is difficult to control for the effect of previous RSV exposure in conducting analysis on responses to RSV vaccination, as all participants will have differing degrees of protection against RSV, which will make it more difficult to understand the relationship between immune transcriptomic signalling and antibody mediated immunological protection.

This was a post hoc analysis and subject to limitations common to first-in-man clinical trials in that there were only a small number of volunteers in each prime/boost study group and the potential for confounding chance observations. This included insufficient statistical power for direct between-group comparisons. The within group analyses used here were applied with all effort to correct for baseline heterogeneity using the *lmfit* model, but we cannot assume that differences in the magnitude of gene expression was driven by vaccine alone. For example, more differentially expressed probes were detected 3 days after IM PanAd3-RSV in study group 2 (compared with study group one), when 6/10 group 2 individuals were actually sampled 2 days after vaccination and all other samples were obtained 3 days after vaccination (this was permissible in the trial protocol). Adding the day of sampling as well as the baseline serum PanAd3 (vector) neutralizing antibody titre, which was also significantly different between study groups 1 and 2 volunteers, as covariates into the *lmfit* model did not significantly alter the number of differentially expressed probes following IM PanAd3-RSV prime (data not shown). Using DNA microarray data from whole blood is representative of the gene expression from a diversity of cell populations in circulation. What remains unclear is the extent to which the fold-change in gene expression from whole blood is influenced by relative changes in different cell populations after vaccination. For example, neutrophils transiently declined 3 days after vaccination (data not shown) which was not included in the *lmfit* model.

The findings of this study should be confirmed in further studies. The capacity to predict neutralizing antibody responses should ideally be modelled in a controlled study designed specifically to measure the relationship between expression of these genes and neutralization. This could be done in further mouse and human experiments where participants are vaccinated with RSV001, and expression of specific genes are assessed in a controlled study designed to determine the relationship between expression of these genes and a neutralization response. It would also be interesting to assess if the protein expression of the genes identified in this study are also associated with neutralization via an assay that could quantify protein expression, such as western blot. This would allow us to assess if the effects on neutralization of these genes is due to some downstream genes regulated by the genes identified in this study, or by expression of the identified genes themselves.

It is difficult with this study design to determine the efficacy of the vaccine in the populations studied, as rates of infection after vaccination were not tested. While it is possible in this study to compare gene expression to markers of vaccine efficacy, such as neutralization and expression of markers such as interferons, there is not yet a single widely accepted correlate for protection against RSV. Testing for relationship of this biomarker with protective efficacy of the vaccine would require a study where infection rates of each participant are tracked over an RSV season and rate at which RSV infections are developed, as well as the severity of those infections is tracked and compared with gene expression markers.

As PanAd3 and MVA constructs without the RSV insert were not included in this study, it was impossible to make the comparison between the response to the vector and the more specific responses to the insert.

## Conclusions

A small, shared core of transcriptional responses 3 days after vaccination, mostly related to type I interferon responses typically followed each dose of IM vaccine, with MVA responsible for a greater magnitude of transcriptional changes than the adenovirus vector. Analysis of the gene expression profiles has revealed the potential for expression of discrete and inter-dependent molecular pathways in response to different route of immunisation and different viral vectors.

## Supplementary Material

uxad003_suppl_Supplementary_Material

## Abbreviations

DEGs: differentially expressed genes
GO: gene ontology
GSEA: gene set enrichment analysis
IFN: interferon
IM: intramuscular
IN: intranasal
MVA: modified vaccinia ankara
PanAd: Chimpanzee adenovirus
RSV: respiratory syncytial virus.

## Data and materials availability

The RSV001 clinical trial was conducted in accordance with the UK Medicines for Human Use (Clinical Trials) Regulations 2004, and subsequent amendment. RSV001 was registered with clinicaltrials.gov (reference NCT01805921), the European Medicines Agency’s European Clinical Trials Database (EudraCT; reference 2011-003589-34) and was portfolio adopted by the National Institute for Health Clinical Research Network. The following regulatory approvals were granted before study procedures commenced.

• Clinical Trials Authorisation from the UK Medicines and Healthcare Products Regulatory Agency (MHRA reference 35082/0003/001-0001)

• University of Oxford Genetic Modification Safety Committee (GSMC reference GM462.11.64)

• Research Ethics Committee (NRES Berkshire reference 13/SC/0023)

• Local site and NHS R&D approvals
