# Supplementary material for: Transcriptomic response and immunological responses to chimpanzee adenovirus- and MVA viral-vectored vaccines for RSV in healthy adults
Source: Clin Exp Immunol. 2023 Jan 9;211(3):269–79. doi: 10.1093/cei/uxad003 (PMC10038321; doi:10.1093/cei/uxad003)
Supplement: uxad003_suppl_Supplementary_Material [file uxad003_suppl_supplementary_material.docx]

# Supplemental methods

## Sample processing

Total RNA was extracted from 3mL of whole blood (Tempus^TM^ Spin RNA Isolation Kit; Applied Biosystems, Life Technologies, California, USA) collected in Tempus RNA^TM^ tubes (Applied Biosystems, UK) immediately before, three and seven-days after each dose of vaccine was given from every volunteer and stored at -80**°**C for later analysis. Extraction was performed with an on-column DNA digestion and elimination of α- and β-globin mRNA using magnetic bead separation and the GLOBINclear^TM^ Kit (Ambion, Life Technologies).

## Vaccine constructs

The construction and pre-clinical evaluation of these genetically modified organisms were described previously*(17)(18)(19)*. Briefly, the PanAd3 vector was derived from *Pan paniscus* chimpanzees and was rendered replication-defective by deletion of the E1 and E4 loci and used here for the first time (first-in-man vector). Modified vaccinia virus Ankara, or MVA, which cannot naturally replicate in mammalian cells, has been used extensively in vaccine trials for other diseases and cancer, and has shown promising results in smallpox*(20)*. Each vector was genetically modified for use as vaccine with insertion of the same single fragment of codon-optimised DNA to express three RSV antigens, under the control of a CMV promoter. These included a modified fusion protein (deleted transmembrane region, F0ΔTM) to be released as a soluble antigen and two intra-cellular antigens, of RSV,the nucleocapsid (N) and matrix (M2-1) proteins joined by a flexible linker region.

## Volunteers/Clinical trial

The RSV001 clinical trial was an open-label, dose-escalation, phase one evaluation of safety and immunogenicity of four combinations of prime/boost PanAd3-RSV and MVA-RSV vaccine, using combinations of vaccine in 42 healthy adults aged 18-50 years, with the primary findings from the study previously published*(21)*. Volunteers were randomized into four groups and were primed with either intramuscular (IM) or intra-nasal (IN) PanAd3-RSV and 4- or 8-weeks later boosted with either IM PanAd3-RSV or IM MVA-RSV (Table. 1). A further group of 18 older adults aged 65-75 years was given the same combinations of prime and boost vaccines, with an additional naïve group of 6 participants*(22)*.

## Data processing

Quality control and gene expression analyses used the Bioconductor platform in R Studio (Version 3.1.3). Raw fluorescent Illumina probe intensities were read into R (Version 3.5.3) using the ‘read.ilmn’ function of the *limma* package (version 3.44.1)*(24)*. Data quality was assessed using the ‘arrayQualitymetrics’ package (3.4.1)*(25)*. The data were quantile normalized, background subtracted and log2-transformed using the neqc() function in limma. Probes with a detection p-value > 0.05 in more than 90% of the participants, without a gene symbol or map to the human genome were excluded*(26)*.

Statistical correlations with clinical and immunological responses to vaccination

Differentially expressed probes 3-days and 7-days after vaccination (FDR < 0.05) were used in the analysis of transcriptomic correlates of vaccine responses measured at later time points. The log_2_ fold change of each probe for each volunteer was correlated with pre-selected individual immune responses to vaccination (namely the fold-change in serum RSV and vector neutralising antibody titres, IFN-γ T cell responses in response to RSV F protein, and F-specific antibody secreting cell responses) and then filtered for statistical significance (Pearson’s test, p-value <0.05). These antibody, cell and cytokine data have been previously published*(22)* alongside their respective laboratory methods.

CIBERSORTx deconvolution of transcriptomics data

CIBERSORTx is an algorithm that deconvolutes transcriptomics data by comparison of the gene expression profile to a number of pure expression profiles for selected cell types*(31)*, in order to give an estimate of cell populations being induced in the samples. The CIBERSORTx algorithm (version 1.05) was given a matrix of all genes contained on the microarray with gene symbols given in Human Genome Organisation (HUGO). A reference file (LM22) provided to use with the CIBERSORTx algorithm was used as a cell type reference. The algorithm was run over 500 iterations and results plotted using the ggplot2 package (version 3.3.1)*(32)* in R.

## Real time qPCR in older adults

Relevant genes were selected from the set of differentially expressed genes based on statistical correlations with clinical outcomes, high levels of expression in response to vaccination and roles in vaccine responses and type I regulatory networks based on literature for confirmation by qPCR in the older adult group. RNA was extracted from Tempus blood tubes for day zero and day three post vaccination and at day three post boost. Changes in expression of the genes *IFI27, RSAD2, IFIT3, MX1, ATF3 and OAS1 (*all found to be differentially expressed in microarray analyses) in response to vaccination were assessed by qPCR with SYBR green dye using a StepOnePlus machine. *Β-actin* and *GAPDH* were selected to use as housekeeping genes. Fold changes in gene expression were calculated using the 2^-δδCT^ method*(37)*. Gene expression was compared between day of vaccination and days 3 and 7 post vaccination.


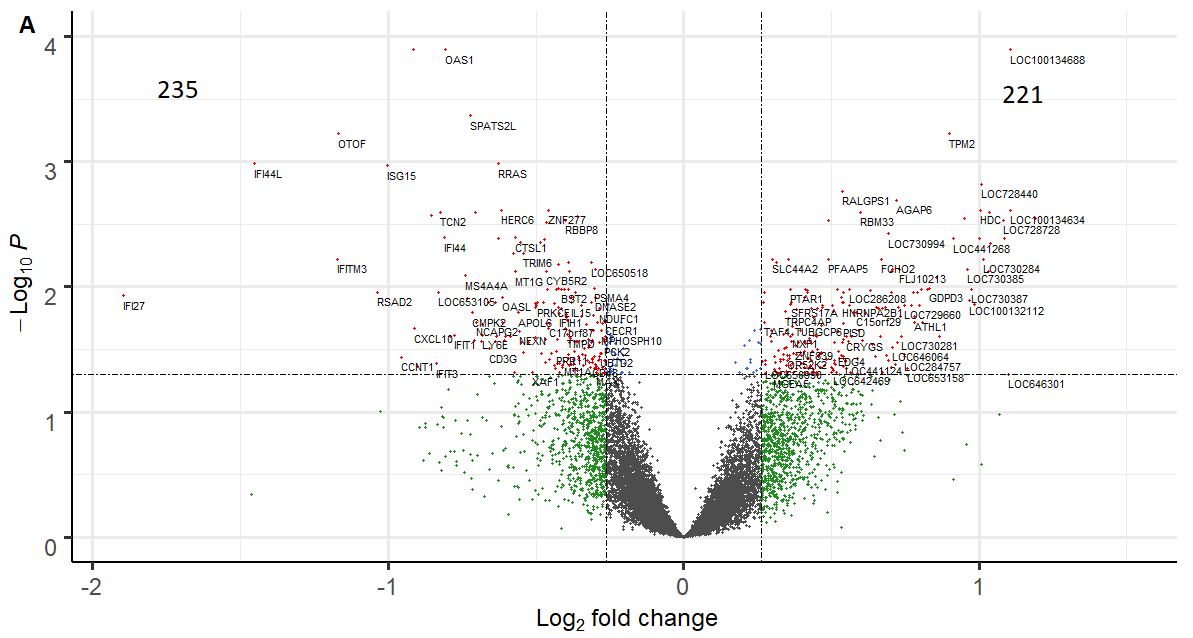


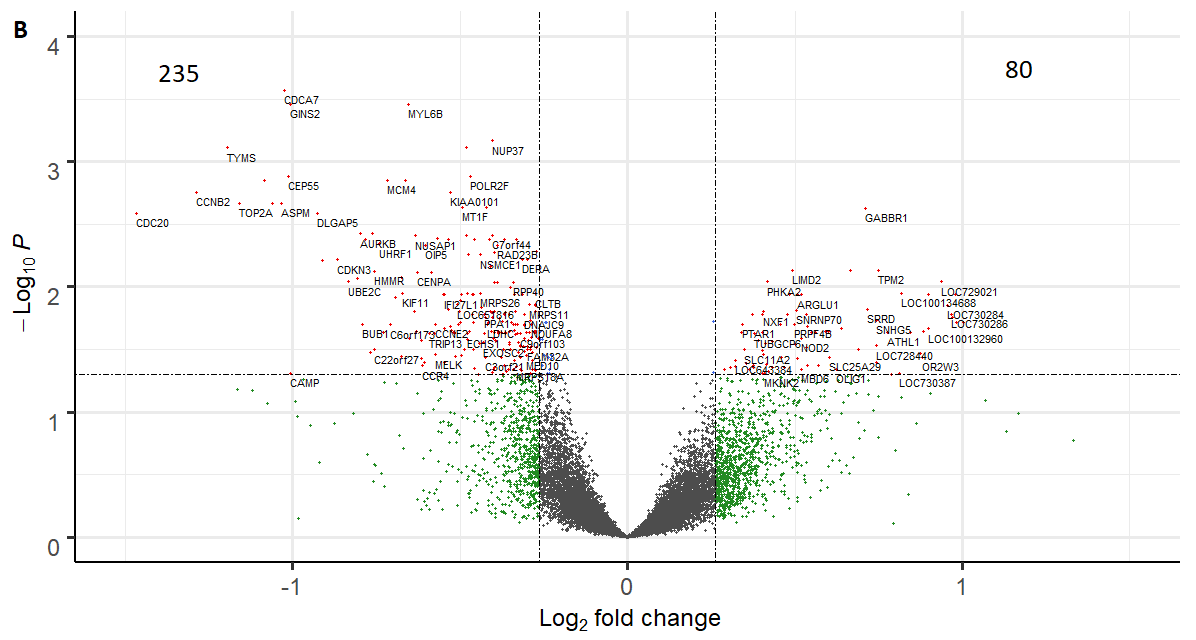


sFig. 1. Volcano plot illustration of differential expression between prime and boost vaccination at A – 3 days and B – 7 days post vaccination for participants primed intramuscularly and participants primed intranasally and boosted intramuscularly N=10 volunteers who received low-dose and target-dose MVA-RSV boost vaccination by IM injection after IN priming with PanAd3-RSV. N=21 volunteers who received intramuscular PanAd3-RSV. The difference in transcription between prime and boost vaccination is shown. Left side of plot indicates higher expression in prime vaccination while right side indicates higher expression in boost vaccination. Red dots denote significantly differentially expressed probes (adj.p.val <0.05, fold change > 1.2), blue dots significantly differentially expressed probes with fold change < 1.2, green dots indicate genes with a fold change > 1.2 but which are not significantly differentially expressed, grey dots indicate probes that are not significantly differentially expressed. Horizontal dotted lines represent FDR cutoffs, vertical dotted lines represent log2 fold change cutoffs.


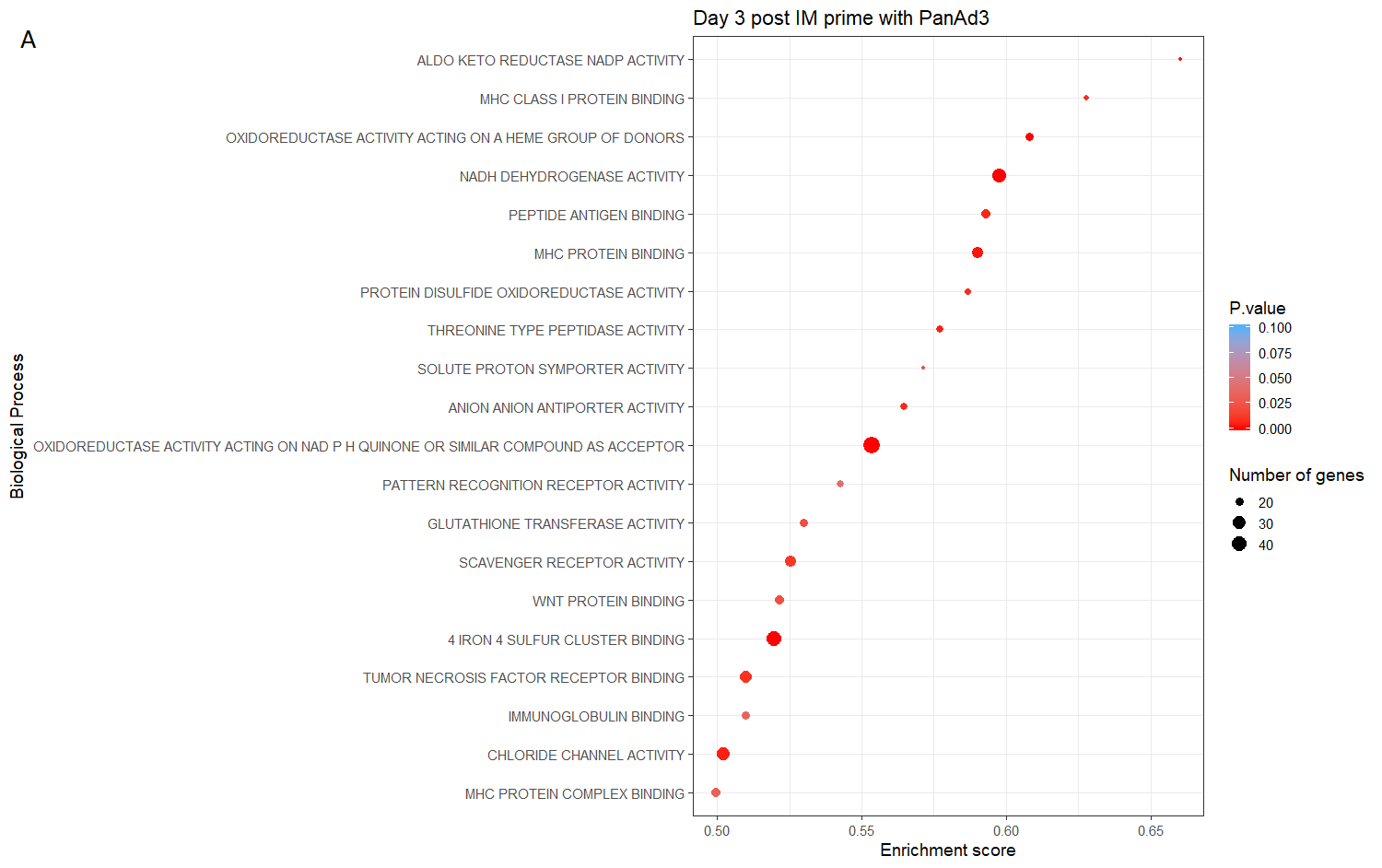


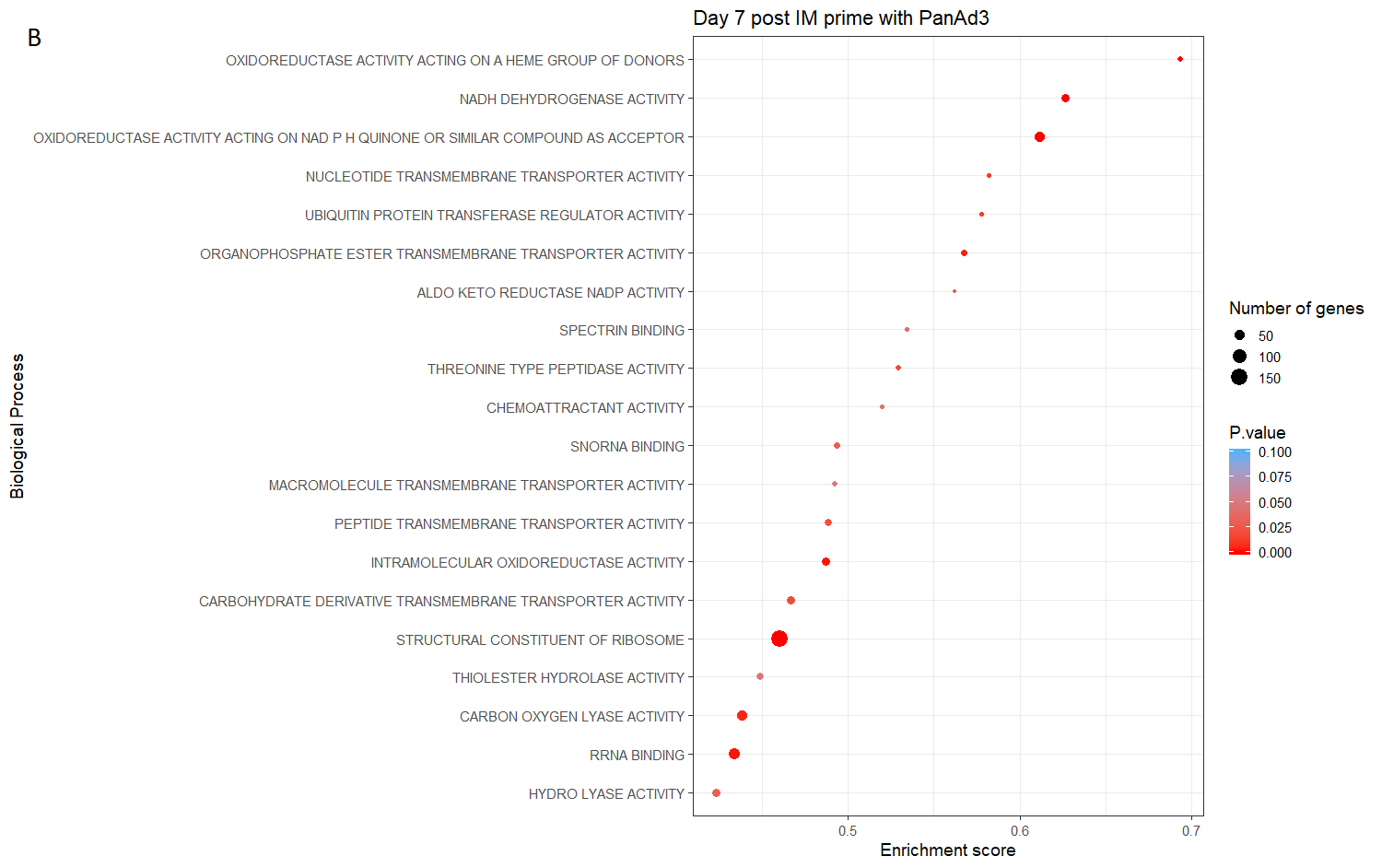


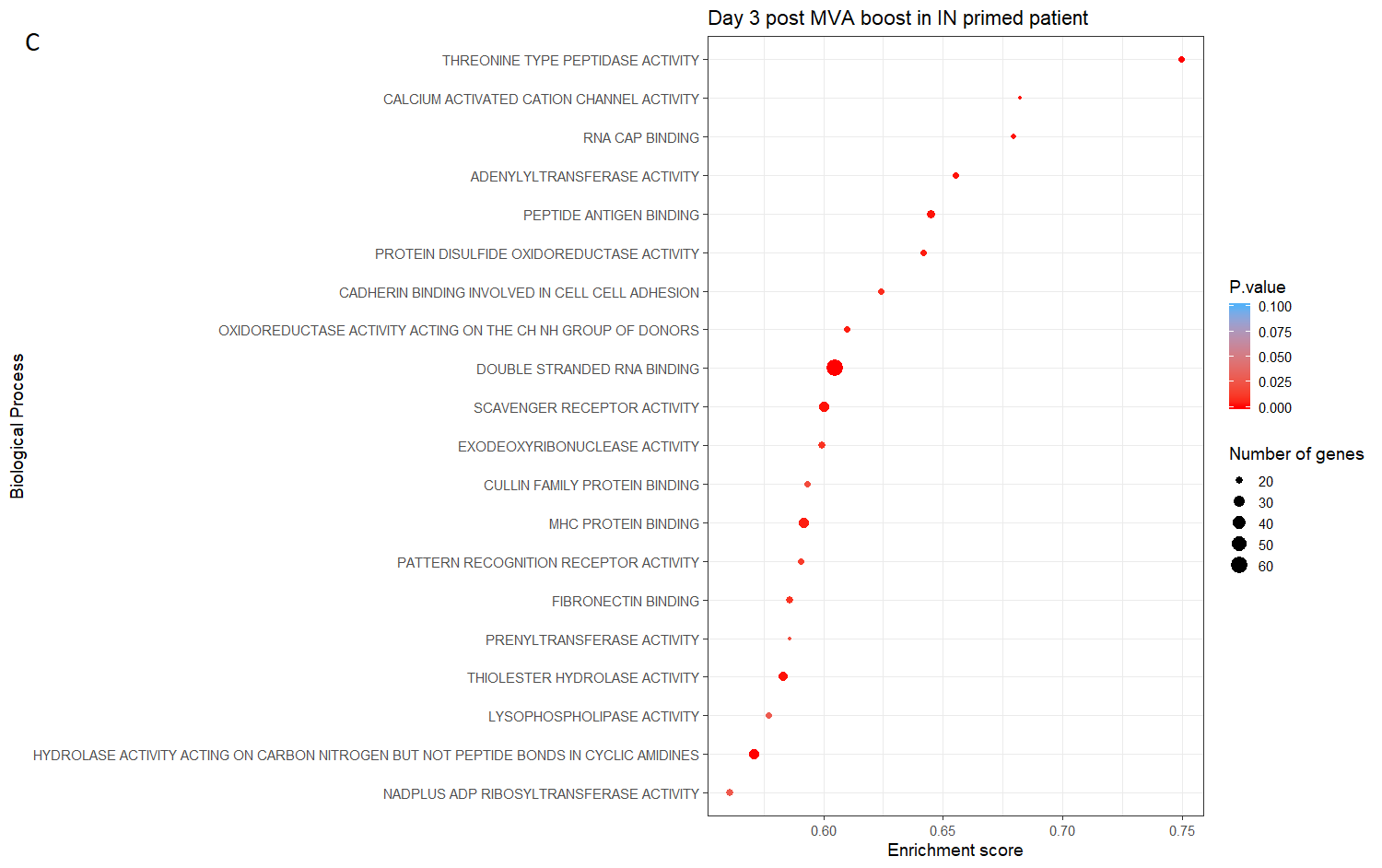


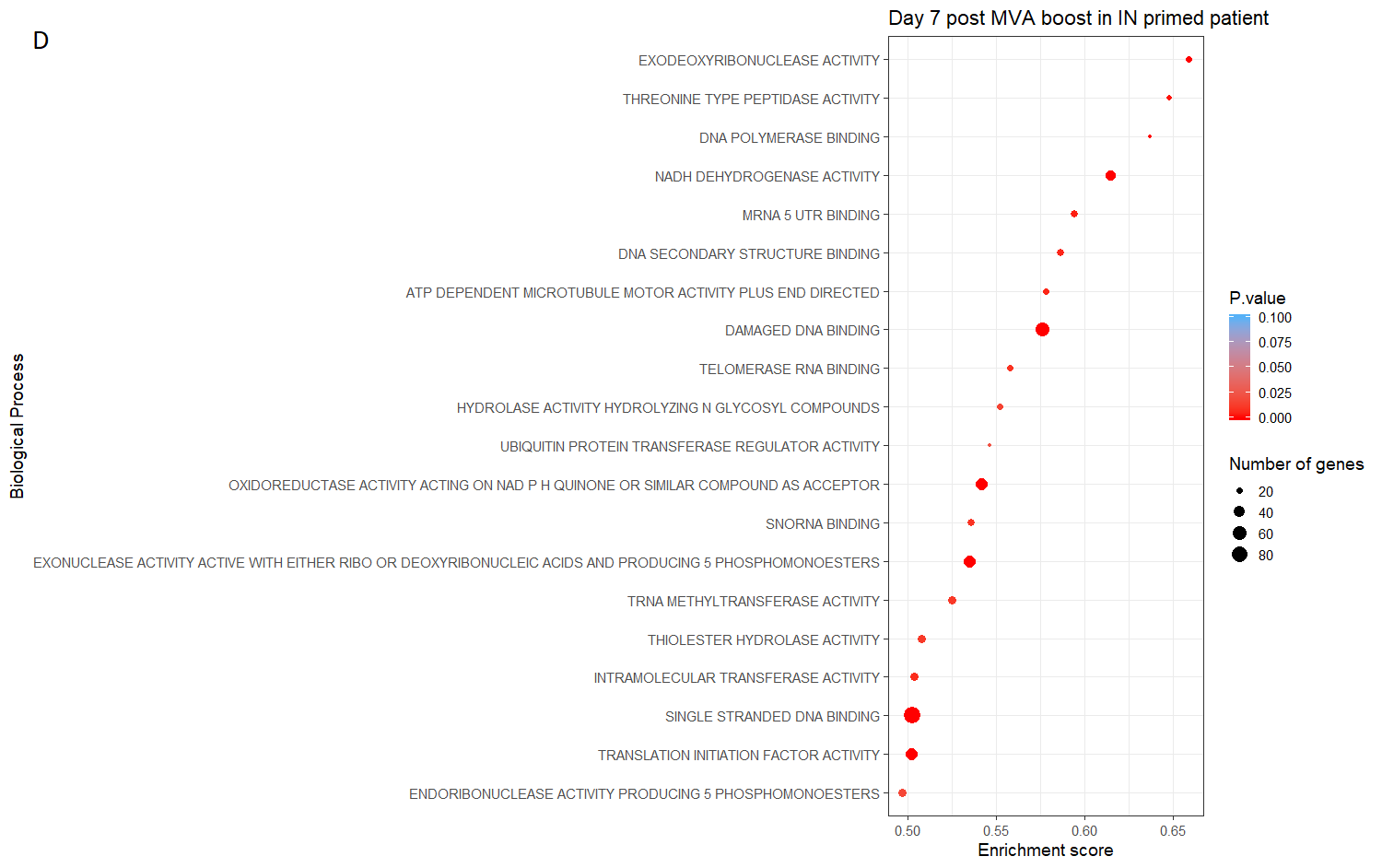


sFig. 2. Gene set enrichment analysis of molecular function terms of the response to prime vaccination three and seven days after vaccination

Gene enrichment analysis of molecular function terms was performed on the ranked list of all probes on the array at three (A) and seven days (B) post prime vaccination and three (C) and seven days (D) post boost vaccination by MVA-RSV in participants primed intranasally. Most significantly enriched terms are ranked by enrichment score. Dots are coloured based on the FDR of the associated term. Size of dots is based on the number of genes associated with that term. FDR <0.05 was used.


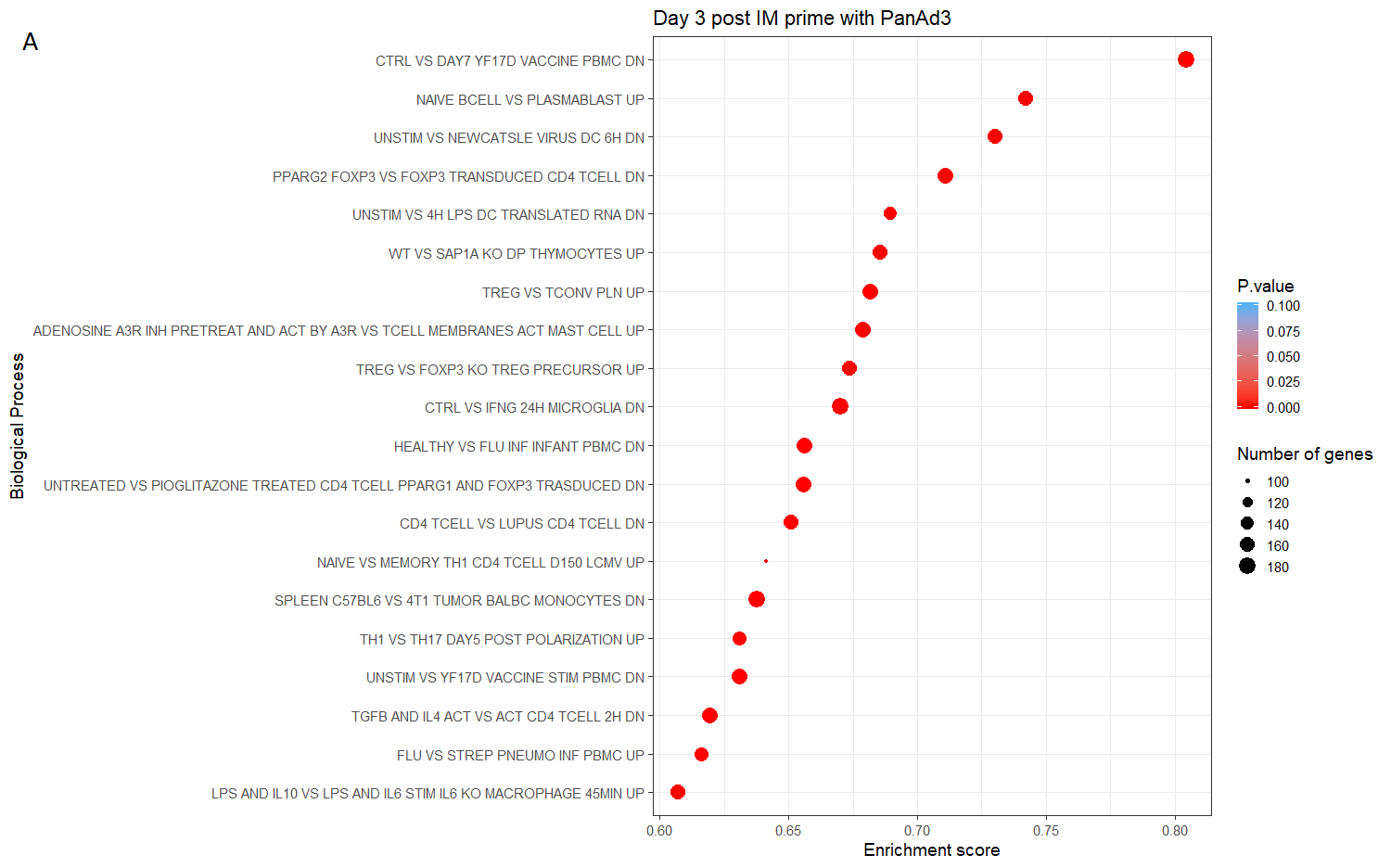


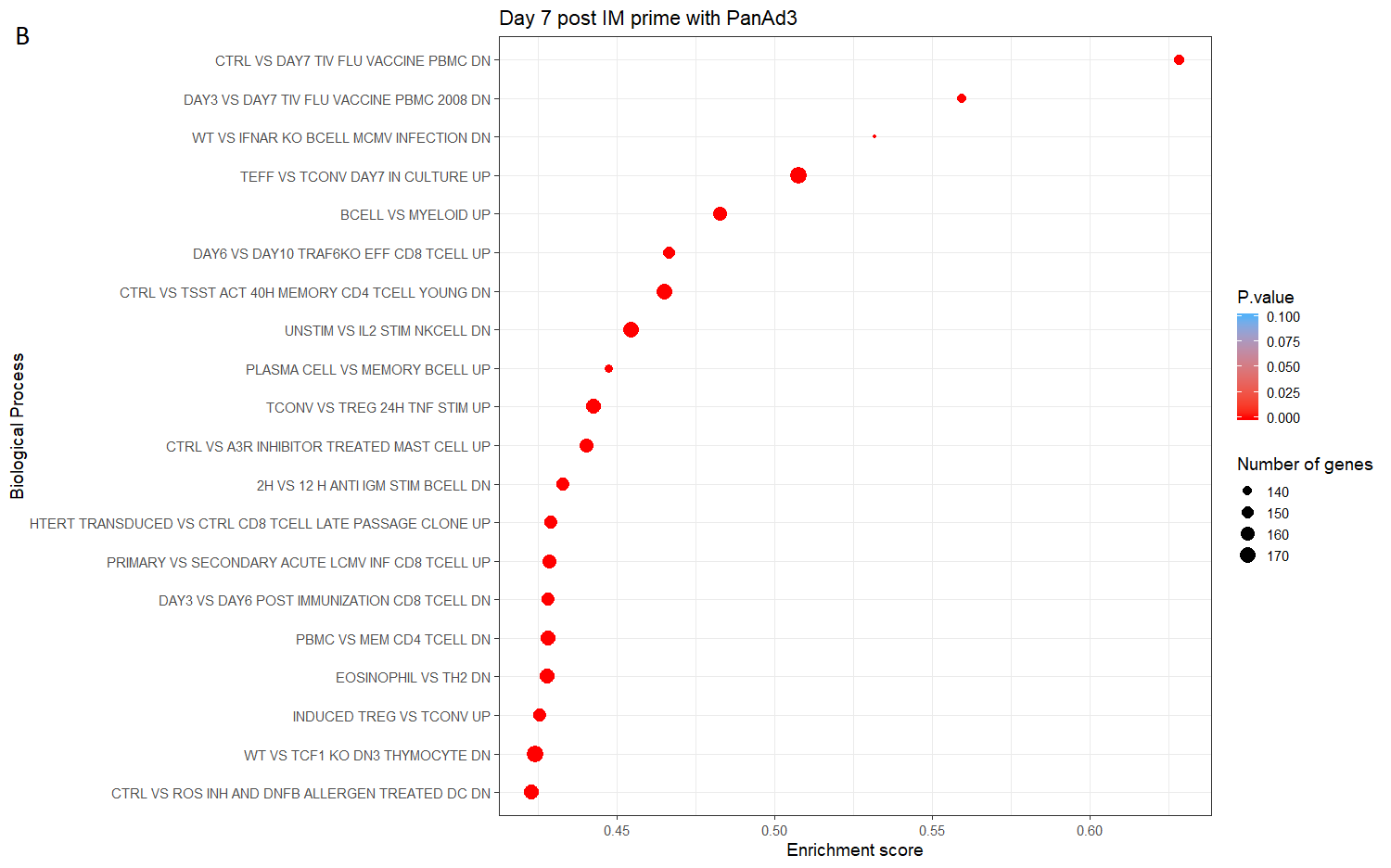


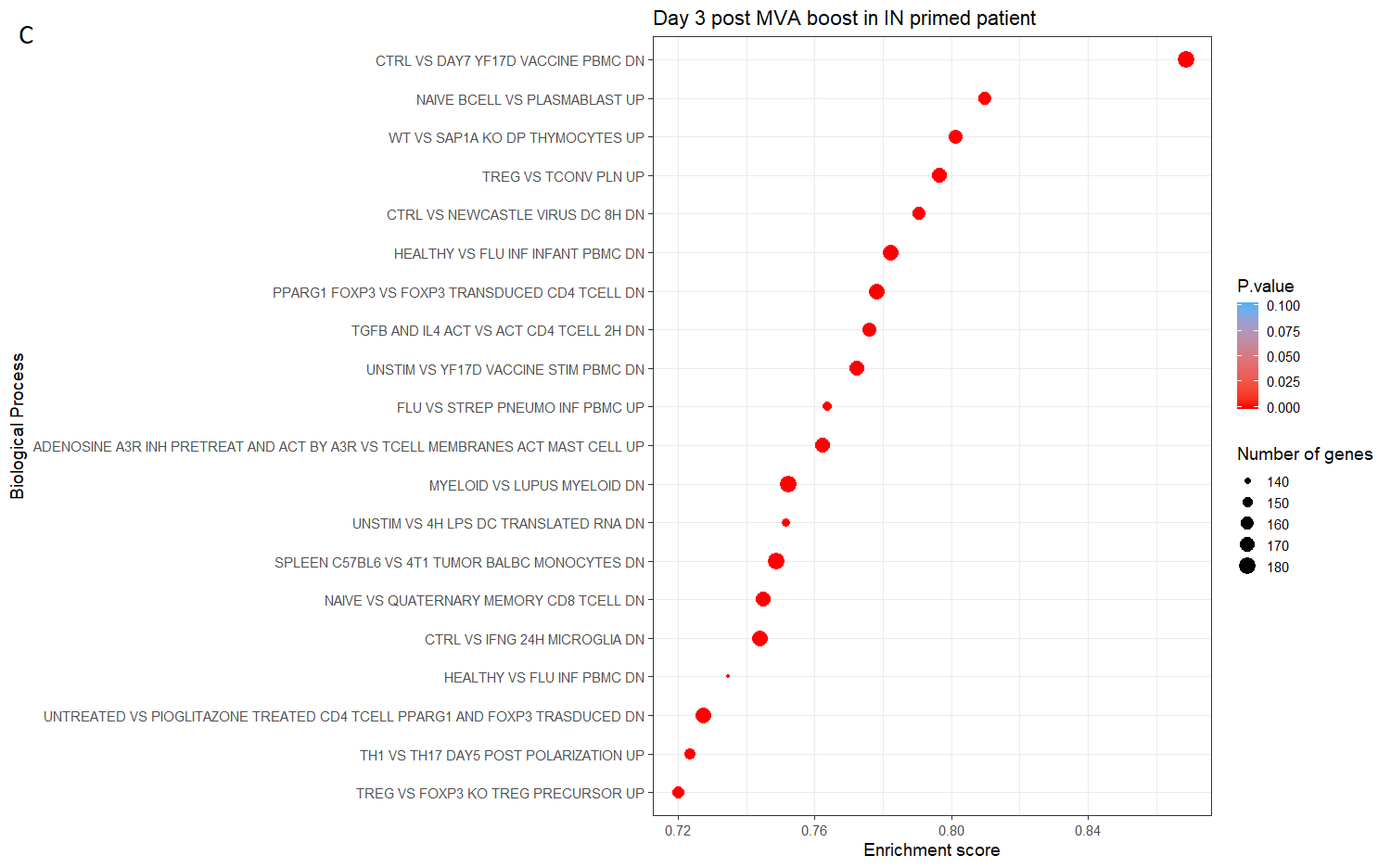


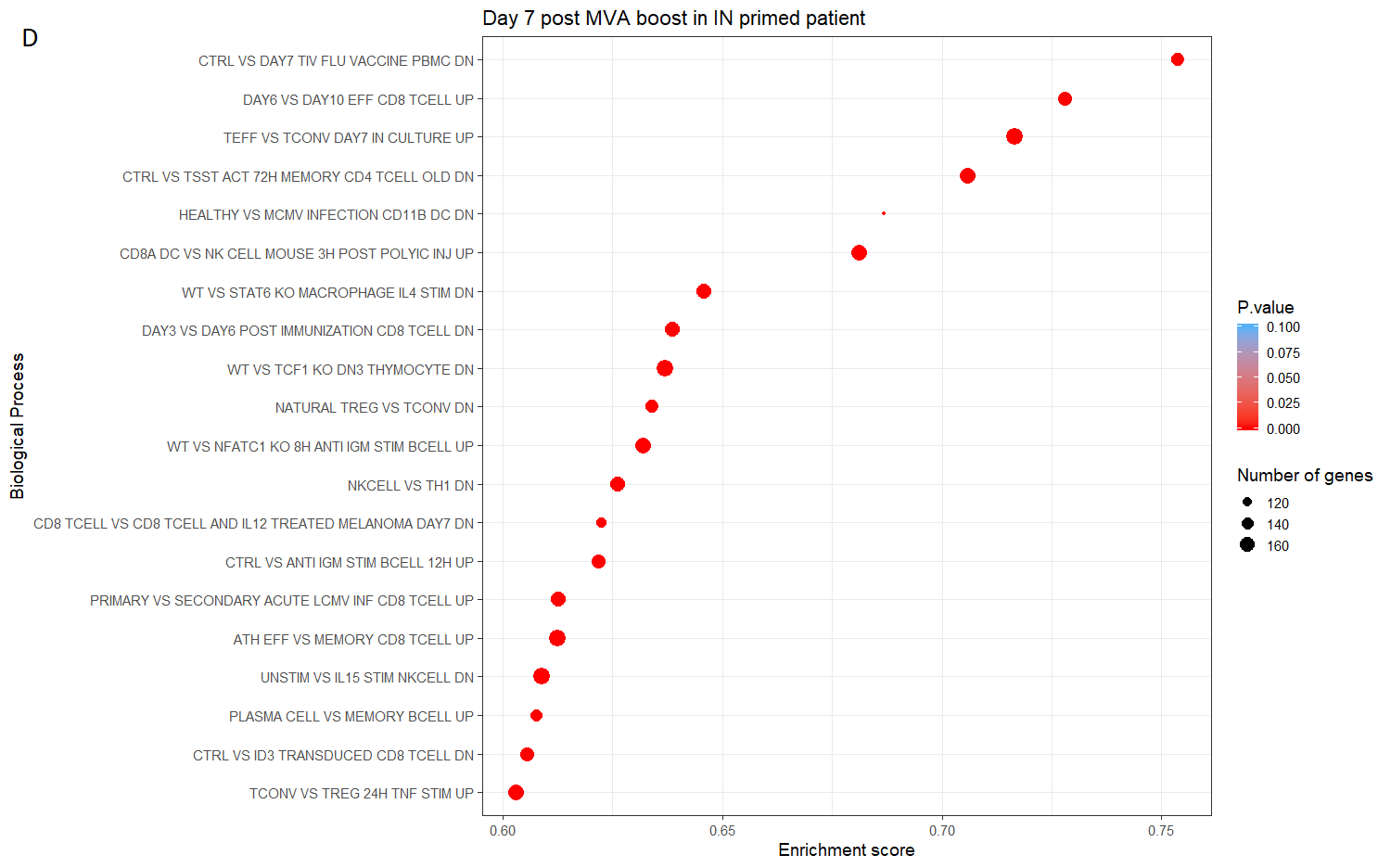


sFig. 3. Gene set enrichment analysis of immunological terms of the response to prime vaccination three and seven days after vaccination

Gene enrichment analysis of immunological terms was performed on the ranked list of all probes on the array at three (A) and seven days (B) post prime vaccination and three (C) and seven days (D) post boost vaccination by MVA-RSV in participants primed intranasally. Most significantly enriched terms are ranked by enrichment score. Dots are coloured based on the FDR of the associated term. Size of dots is based on the number of genes associated with that term. FDR <0.05 was used.

| Vector Neutralising antibody IM prime | | |
| --- | --- | --- |
| GeneSymbol | Correlation | P Value |
| IFI44L | 0.458819137 | 0.041867318 |
| DHX58 | 0.478375363 | 0.032875213 |
| OAS1 | 0.4655886 | 0.038562452 |
| FLJ14166 | -0.511470514 | 0.021163843 |

sTable 1. Statistically significant correlations between transcriptional changes 3-days after IM PanAd3-RSV prime and changes in serum titres of anti-PanAd3 (vector) neutralising antibody measured 4- and 8-weeks after vaccination.

The log_2_-fold change in gene expression from each differentially expressed probe 3-days after vaccination was correlated by Pearson’s correlation with the log_2_ transformed fold-change in serum anti-PanAd3 (vector) neutralising antibody measured 28-days after prime or boost vaccination. Correlations with P <0.05 are shown. Serum vector neutralising antibody titres were not recorded after boost vaccination.

| Neutralising antibody prime | | | | | |
| --- | --- | --- | --- | --- | --- |
| Gene Symbol | | Correlation | | | P. value |
| OASL | | 0.49924812 | | | 0.026557555 |
| ATF3 | | 0.485714286 | | | 0.031475535 |
| IFI35 | | 0.476691729 | | | 0.035138699 |
| GCH1 | | 0.74887218 | | | 0.000220568 |
| SIDT2 | | 0.494736842 | | | 0.028123156 |
| TAP1 | | 0.542857143 | | | 0.014730928 |
| C17orf87 | | 0.502255639 | | | 0.025553189 |
| MT1A | | 0.455639098 | | | 0.045004489 |
| FER1L3 | | 0.490225564 | | | 0.029761695 |
| FBXO6 | | 0.640601504 | | | 0.002942956 |
| WARS | | 0.590977444 | | | 0.007060087 |
| GBP1 | | 0.487218045 | | | 0.030895741 |
| VAMP5 | | 0.496240602 | | | 0.0275933 |
| LOC731486 | | 0.512781955 | | | 0.022275223 |
| BATF3 | | 0.497744361 | | | 0.027071462 |
| LILRB1 | | 0.521804511 | | | 0.019743148 |
| GBP4 | | 0.536842105 | | | 0.016043222 |
| ECGF1 | | 0.455639098 | | | 0.045004489 |
| NOTCH2NL | | -0.527819549 | | | 0.018188281 |
| OAS1 | | 0.472180451 | | | 0.037092882 |
| CSTF3 | | -0.461654135 | | | 0.041987538 |
| C11orf75 | | 0.454135338 | | | 0.04578466 |
| WARS | | 0.455639098 | | | 0.045004489 |
| IFIT2 | | 0.508270677 | | | 0.023635872 |
| EPB41L3 | | 0.506766917 | | | 0.024103986 |
| MUC1 | | 0.532330827 | | | 0.017088471 |
| TLR7 | | 0.508270677 | | | 0.023635872 |
| GRAMD1B | | 0.446616541 | | | 0.049845818 |
| CDK10 | | -0.496240602 | | | 0.0275933 |
| ENO3 | | -0.466165414 | | | 0.039831128 |
| CD24 | | 0.476691729 | | | 0.035138699 |
| FBP1 | | 0.541353383 | | | 0.015050505 |
| TLE1 | | 0.491729323 | | | 0.029207266 |
| LILRB4 | | 0.497744361 | | | 0.027071462 |
| RTN3 | | 0.530827068 | | | 0.017448911 |
| Neutralising antibody IM prime IM boost | | | | | |
| Gene Symbol | Correlation | | | P Value | |
| CYSLTR1 | 0.716666667 | | | 0.036866182 | |
| IFI35 | 0.766666667 | | | 0.021389991 | |
| LILRB4 | 0.833333333 | | | 0.008267196 | |
| RTP4 | 0.716666667 | | | 0.036866182 | |
| SLC2A6 | 0.8 | | | 0.013828263 | |
| TRIM6 | -0.916666667 | | | 0.001311728 | |
| Neutralising antibody IN prime IM boost | | | | | |
| Gene Symbol | Correlation | | P value | | |
| CMPK2 | -0.783333333 | | 0.017223325 | | |
| BLVRA | -0.833333333 | | 0.008267196 | | |
| ADAP2 | -0.9 | | 0.002028219 | | |
| OASL | -0.75 | | 0.02549052 | | |
| TLR7 | -0.7 | | 0.043253968 | | |
| FBXO6 | -0.733333333 | | 0.031123236 | | |
| CD68 | -0.8 | | 0.013828263 | | |
| DHRS9 | -0.783333333 | | 0.017223325 | | |
| SH3RF1 | -0.783333333 | | 0.017223325 | | |
| LMO2 | -0.783333333 | | 0.017223325 | | |
| MARCH1 | -0.75 | | 0.02549052 | | |
| C3AR1 | -0.833333333 | | 0.008267196 | | |
| GRN | -0.75 | | 0.02549052 | | |
| SLC30A1 | -0.783333333 | | 0.017223325 | | |
| ACOT9 | -0.7 | | 0.043253968 | | |
| TYMP | -0.7 | | 0.043253968 | | |
| DDX58 | -0.766666667 | | 0.021389991 | | |
| C17orf58 | -0.833333333 | | 0.008267196 | | |
| HPSE | -0.75 | | 0.02549052 | | |
| ADAMDEC1 | -0.7 | | 0.043253968 | | |
| NIPAL2 | -0.9 | | 0.002028219 | | |
| MARCH1 | -0.716666667 | | 0.036866182 | | |
| HEY1 | 0.716666667 | | 0.036866182 | | |
| LGALS9 | -0.716666667 | | 0.036866182 | | |
| ACOX2 | -0.766666667 | | 0.021389991 | | |
| RNH1 | -0.75 | | 0.02549052 | | |
| CCL2 | -0.716666667 | | 0.036866182 | | |
| LOC100128191 | -0.866666667 | | 0.004508377 | | |
| NR1H3 | -0.7 | | 0.043253968 | | |
| NBPF1 | -0.716666667 | | 0.036866182 | | |
| TNFRSF1B | -0.816666667 | | 0.0107694 | | |
| DAB2 | 0.783333333 | | 0.017223325 | | |

sTable 2. Statistically significant correlations between transcriptional changes 3-days after vaccination and changes serum RSV-neutralising antibody titres 28-days after vaccination.

The log_2_-fold change in gene expression from each differentially expressed probe 3-days after vaccination was correlated by Pearson’s correlation with the log_2_ fold-change in serum RSV-neutralising antibody titre 28-days after vaccination. Correlations with P < 0.05 are shown.

| Antibody secreting cells – IgA IM prime | | |
| --- | --- | --- |
| GeneSymbol | Correlation | P Value |
| IFI27 | 0.425889 | 0.043926 |
| OAS1 | 0.442688 | 0.035628 |
| RGS12 | 0.442688 | 0.035628 |
| HES4 | 0.488142 | 0.019296 |
| CTSL1 | 0.464427 | 0.026808 |
| IRS2 | -0.45949 | 0.028638 |
| TP53INP2 | -0.49209 | 0.018231 |
| CSTF3 | 0.442688 | 0.035628 |
| OTOF | 0.450593 | 0.032185 |
| CSTF3 | 0.528656 | 0.010477 |
| CCNJL | -0.43083 | 0.04134 |
| IL1R2 | -0.4753 | 0.023115 |
| PPAP2C | -0.4753 | 0.023115 |
| MPZL1 | -0.42292 | 0.045538 |
| KDM6B | -0.62451 | 0.001819 |
| TMOD2 | 0.481225 | 0.021282 |
| SNORD99 | 0.505929 | 0.014876 |
| ORM1 | -0.43775 | 0.037926 |
| CNOT3 | -0.45553 | 0.030173 |
| APOBEC3G | 0.486166 | 0.019847 |
| TMCC3 | -0.55138 | 0.007217 |
| EMR3 | -0.50593 | 0.014876 |
| NECAB2 | -0.51779 | 0.012422 |
| Antibody secreting cells – IgG IM prime | | |
| GeneSymbol | Correlation | P Value |
| C1QB | 0.518429 | 0.033003 |
| OAS3 | 0.484031 | 0.048982 |
| HERC5 | 0.515972 | 0.03399 |
| IFI6 | 0.594596 | 0.011823 |
| TAGLN | 0.565112 | 0.018087 |
| DHX58 | 0.636366 | 0.006024 |
| TAP1 | 0.484031 | 0.048982 |
| FGD2 | 0.587225 | 0.013196 |
| FER1L3 | 0.508601 | 0.037088 |
| MARCO | 0.520886 | 0.032038 |
| MS4A14 | 0.572483 | 0.016321 |
| TSPAN4 | 0.562655 | 0.018707 |
| HLA-DRB4 | 0.582311 | 0.01418 |
| C13orf18 | -0.59951 | 0.010972 |
| TRPM4 | 0.496316 | 0.042722 |
| SLC43A3 | 0.498773 | 0.041547 |
| MTMR11 | 0.496316 | 0.042722 |
| CDKN1A | 0.547913 | 0.02279 |
| RTN3 | -0.48894 | 0.0464 |
| CD79A | -0.50123 | 0.040396 |
| ASCA IN prime IM boost | | |
| GeneSymbol | ASCA_F_Corr | ASCA_F_Pval |
| LOC91561 | -0.4614625 | 0.027893837 |
| POFUT2 | 0.41699605 | 0.048901822 |
| CLEC9A | -0.465415 | 0.026453349 |
| RPS6KA5 | 0.42588933 | 0.043925663 |
| FLJ33590 | 0.42687747 | 0.043398564 |
| CAPRIN2 | 0.4298419 | 0.041847377 |
| BTBD3 | 0.46541502 | 0.026453349 |
| VASP | 0.43774704 | 0.037925903 |
| ZNF550 | 0.45652174 | 0.029783077 |
| ASCG IN prime IM boost | | |
| GeneSymbol | ASCA_F_Corr | ASCA_F_Pval |
| LOC91561 | -0.51128 | 0.022722 |
| SAMHD1 | -0.49624 | 0.027593 |
| LOC158345 | -0.55188 | 0.012927 |
| LOC441013 | -0.61805 | 0.004454 |
| LOC401321 | -0.49774 | 0.027071 |
| WDR35 | -0.47669 | 0.035139 |

sTable 3. Correlations between transcriptional changes 3-days after vaccination and F-specific IgG and IgA antibody secreting cell response 7-days after vaccination.

The log_2_-fold change in gene expression from each differentially expressed probe 3-days after vaccination was correlated by Pearson’s correlation with the log_2_ anti-F IgG antibody secreting cell (ASC) spot per million PBMCs 7-days after vaccination. Correlations with P <0.05 are shown

| IFNγ correleting genes prime | | |
| --- | --- | --- |
| GeneSymbol | Correlation | P value |
| RRAS | -0.745454545 | 0.018413841 |
| CAMK1D | -0.709090909 | 0.027514119 |
| DHRS9 | -0.890909091 | 0.001380267 |
| FAM156B | 0.660606061 | 0.044026553 |
| RAPGEF2 | -0.660606061 | 0.044026553 |
| LOC100130520 | -0.672727273 | 0.039381409 |
| FCGR1B | -0.709090909 | 0.027514119 |
| BASP1 | -0.660606061 | 0.044026553 |
| SGK1 | -0.684848485 | 0.035091538 |
| DNAJC3 | -0.757575758 | 0.015920829 |
| NAMPT | -0.672727273 | 0.039381409 |
| EMR3 | -0.806060606 | 0.008235571 |
| LOC647030 | -0.745454545 | 0.018413841 |
| HNMT | -0.866666667 | 0.002681415 |
| NOXA1 | 0.648484848 | 0.049042632 |
| IFNg correlating genes IN prime IM boost | | |
| GeneSymbol | IFN_Corr | IFN_ratio_Pval |
| GNS | -0.71667 | 0.036866 |
| LOC642342 | 0.816667 | 0.010769 |
| ABI3 | -0.73333 | 0.031123 |
| SASH1 | -0.76667 | 0.02139 |
| ST3GAL5 | -0.7 | 0.043254 |
| SUSD1 | -0.83333 | 0.008267 |
| GPR137B | -0.91667 | 0.001312 |
| CAST | -0.78333 | 0.017223 |
| TRIM5 | -0.91667 | 0.001312 |
| HLCS | -0.76667 | 0.02139 |
| MYST1 | 0.716667 | 0.036866 |
| FBXL13 | 0.7 | 0.043254 |
| ZDHHC1 | -0.76667 | 0.02139 |
| HIST1H1C | 0.8 | 0.013828 |
| PSMA4 | -0.75 | 0.025491 |
| DUSP1 | -0.75 | 0.025491 |
| C17orf58 | -0.71667 | 0.036866 |
| TAF1B | -0.71667 | 0.036866 |
| ADAMTSL4 | -0.7 | 0.043254 |
| RAPGEFL1 | -0.75 | 0.025491 |

sTable 4. Correlations between transcriptional changes 3-days after vaccination and the T-cell IFNγ response after vaccination.

The log_2_-fold change in gene expression from each differentially expressed probe 3-days after vaccination was correlated by Pearson’s correlation with the log_2_ fold-change T-cell IFNγ spots per million PBMCs measured 14-days after IM PanAd3-RSV prime and 7-days IM MVA-RSV boost vaccination. Correlations with P < 0.05 are shown. There were no significant correlations with the IFNγ response following IM MVA-RSV boost from volunteers primed with IM PanAd3-RSV

| Human primers | | | |
| --- | --- | --- | --- |
| *GAPDH* - Forward | 5’ | TCACCACCATGGAGAAGGC | 3’ |
| *GAPDH* - Reverse | 5’ | GCTAAGCAGTTGGTGGTGCA | 3’ |
| *MX1* - Forward | 5’ | GGCTGTTTACCAGACTCCGACA | 3’ |
| *MX1* - Reverse | 5’ | CACAAAGCCTGGCAGCTCTCTA | 3’ |
| *IFI27* - Forward | 5’ | CGTCCTCCATAGCAGCCAAGAT | 3’ |
| *IFI27* – Reverse | 5’ | ACCCAATGGAGCCCAGGATGAA | 3’ |
| *IFIT3* – Forward | 5’ | AAATTCTGAGGCAGGCCGTT | 3’ |
| *IFIT3* – Reverse | 5’ | TTTCCCAGAGCCTCGACAAC | 3’ |
| *RSAD2* – Forward | 5’ | GGTGCCTGAATCTAACCAGAAG | 3’ |
| *RSAD2* – Reverse | 5’ | CCACGCCAACATCCAGAATA | 3’ |
| *OAS1* – Forward | 5’ | AGGAAAGGTGCTTCCGAGGTAG | 3’ |
| *OAS1* – Reverse | 5’ | GGACTGAGGAAGACAACCAGGT | 3’ |
| *ATF3* – Forward | 5’ | GAGGATTTTGCTAACCTGACACC | 3’ |
| *ATF3* – Reverse | 5’ | TTGACGGTAACTGACTCCAGC | 3’ |

***sTable 5. primers in used RT-qPCR of human samples***

*Forward and reverse primers are shown from 5’ end to 3’*

| *Vaccination* | *DEG on Day 3 post vaccination* | *DEG on Day 3 post vaccination* |
| --- | --- | --- |
| *IM prime vaccination with PanAd3-RSV* | *634* | *12* |
| *IN prime vaccination with PanAd3-RSV* | *0* | *0* |
| *IM Boosting with MVA-RSV after IM priming with PanAd3-RSV* | *12* | *0* |
| *IM boosting with MVA-RSV after IN priming with PanAd3-RSV* | *782* | *36* |

*sTable 6. Description of numbers of differentially expressed genes per contrast at days 3 and 7 post vaccination*


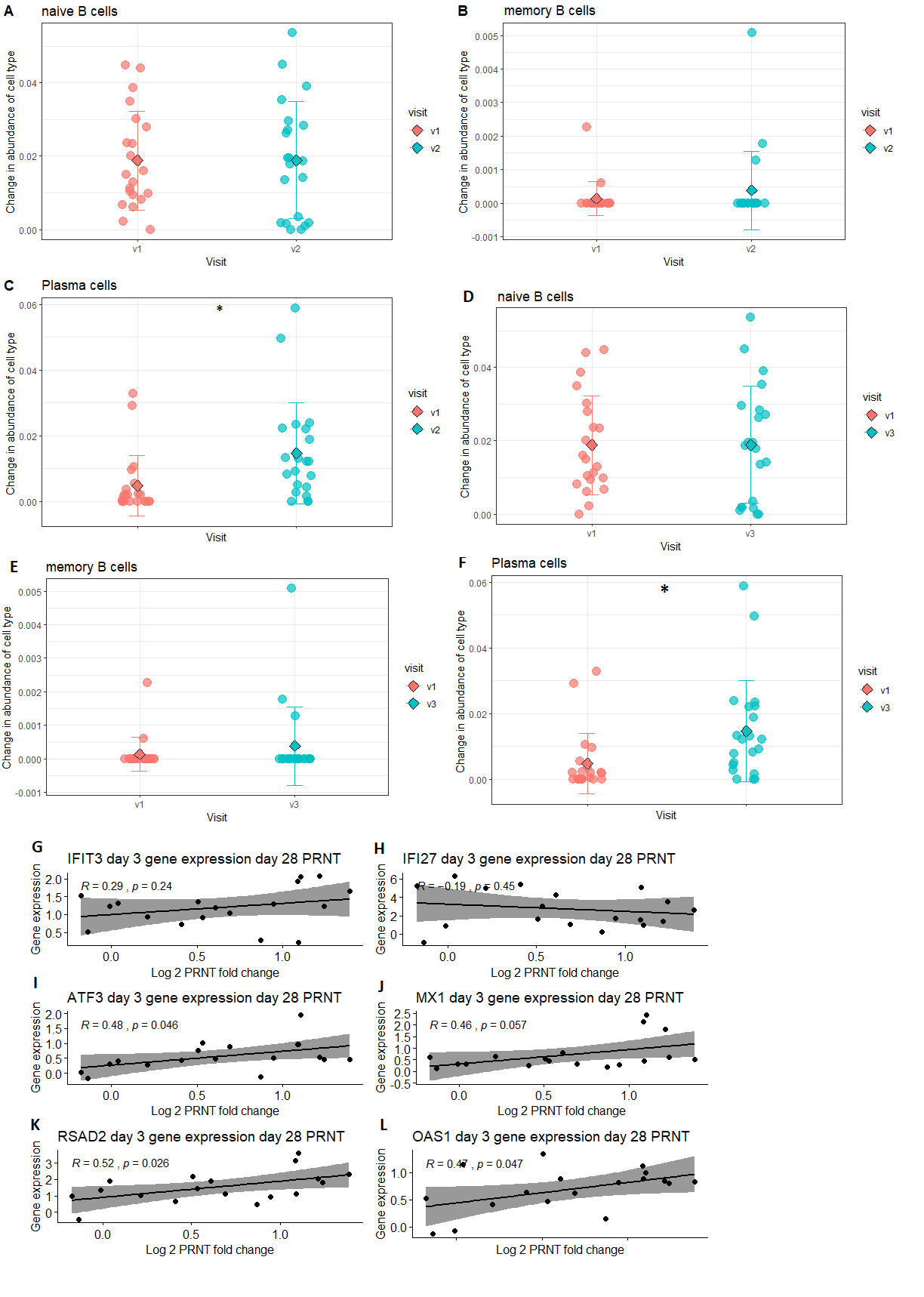


**sFig. 4. (A, B, C) Cibersortx visualisation of B cell and plasma cell abundances at day 0 (v1) and day 3 (v2) post prime vaccination with PanAd3 RSV and (D, E, F) at day 0 and day 7 post prime vaccination with PanAd3 RSV (G-L) Correlations between gene expression in younger adults from microarrays and neutralising antibody** Pearson correlation between changes in gene expression three days post prime vaccination in younger adults as measured by microarray and log 2 fold change in RSV neutralising antibody measured at 28 days post vaccination. Line represents degree of correlation between fold change in gene expression and fold change in neutralising antibody.
